# Development and analysis of a nomogram for predicting pathological response to neoadjuvant immunochemotherapy in locally advanced gastric cancer

**DOI:** 10.3389/fonc.2026.1737833

**Published:** 2026-03-10

**Authors:** Hongyi Yu, Yingjun Pu, Li Wang, Xianfu Li

**Affiliations:** 1 Department of Oncology, Affiliated Hospital of North Sichuan Medical College, Nanchong, Sichuan, China; 2 Department of Radiation Therapy, School of Medical Imaging of North Sichuan Medical College, Nanchong, Sichuan, China; 3 Sichuan Provincial Health Commission Key Medical Laboratory, Nanchong, Sichuan, China

**Keywords:** biomarkers, immunotherapy, locally advanced gastric cancer, major pathological response, neoadjuvant therapy, nomogram

## Abstract

**Background:**

Neoadjuvant immunochemotherapy (NICT) has demonstrated potential to enhance tumor regression in patients with locally advanced gastric cancer (LAGC). However, the benefits for some patients are limited. Existing biological markers have only restricted ability to predict pathological response. New biomarkers and predictive models are essential for identifying patients optimally responsive to immunotherapy.

**Methods:**

In our retrospective analysis, we included LAGC patients who underwent surgical treatment following NICT at our center between January 2021 and March 2025. Classification was done according to the pathological response rates observed in the excised tumor samples, categorizing patients into major pathological response (MPR) and non-MPR groups. Least absolute shrinkage and selection operator (LASSO) regression and multivariable logistic regression models were used to pinpoint risk factors linked to MPR. A nomogram was subsequently constructed using the significant predictors.

**Results:**

In total, 113 LAGC patients fitting the criteria were enrolled, with 46 in the MPR cohort and 67 in the non-MPR cohort, yielding an overall MPR incidence of 40.7%. Independent predictors of MPR following NICT were identified through multivariate logistic regression. These include pre-treatment tumor bed diameter *<* 3.75 cm (OR = 0.22), CEA *<* 1.765 ng/mL (OR = 0.26), CA19-9 *<* 18.390 U/mL (OR = 0.148), NLR *<* 2.422 (OR = 0.265), and SII *<* 597.483 (OR = 0.194). We constructed a nomogram model with an area under the curve of 0.848 (95% CI:0.773–0.923) based on these five predictors. The calibration curve indicated a robust agreement between forecasted probabilities and real MPR occurrences (Hosmer–Lemeshow test: *χ*^2^ = 4.705, 23 P = 0.789).

**Conclusion:**

Tumor bed diameter, CEA, CA19-9, NLR, and SII were determined to be independent predictors of MPR in LAGC patients undergoing NICT. The constructed nomogram demonstrated good accuracy and clinical utility in predicting MPR after NICT, and may help guide the implementation of personalized treatment strategies.

## 1 Introduction

Gastric cancer constitutes the fifth most common malignancy worldwide and the fifth leading cause of cancer mortality (1). In China, it continues to be the third highest cause of cancer-related fatalities (2). Because early-stage gastric cancer often shows few symptoms and screening rates are low, many patients are diagnosed only after the disease has progressed. Locally advanced gastric cancer (LAGC) accounts for nearly 50% to 60% of all cases. Outcomes for these patients are often poor, with fewer than 40% surviving five years after diagnosis (3).

Neoadjuvant therapy is one of the most important perioperative treatment strategies for LAGC. Even after undergoing radical resection followed by adjuvant therapy, these patients still exhibit poor 5-year survival outcomes (4). Consequently, identifying new therapeutic approaches that can extend survival is of substantial importance. Immune checkpoint inhibitors (ICIs) now see widespread application across diverse cancer treatments following recent years of expanded usage, showing a remarkable effect in clinical settings. This advancement has been especially notable in treating head and neck tumors and non-small cell lung cancer (5, 6). When combined with chemotherapy, ICIs may enhance tumor downstaging, eradicate micrometastases, reduce intraoperative dissemination, and lower the likelihood of postoperative recurrence, thereby potentially improving the prognosis for LAGC patients. The KEYNOTE-585 phase III clinical trial confirmed that pairing neoadjuvant chemotherapy with immunotherapy yielded a substantially greater pathological complete response rate, blowing chemotherapy alone out of the water with a striking 12.9% versus a mere 2.0% (p*<*0.00001) (7). Similarly, the DANTE/IKF-s633 trial demonstrated that neoadjuvant immunochemotherapy (NICT) was associated with superior tumor downstaging outcomes. Combining atezolizumab with FLOT increased pCR rates for resectable gastric or gastroesophageal junction (G/GEJ) cancer compared to FLOT alone (23% vs. 15%) (8). Furthermore, Yuan et al. noted a substantial enhancement in the pCR rate in the NEOSUMMIT-01 Phase II study due to the inclusion of toripalimab with perioperative chemotherapy (9).

While immunotherapy helps many patients, it proves ineffective for some and may cause serious side effects and considerable treatment costs (10). Hence, pinpointing patients with the highest potential for positive ICI responses is crucial. Immunotherapy outcomes often prove more successful when specific molecular indicators are present, including microsatellite instability (MSI), defective mismatch repair (MMR) systems, elevated programmed death-ligand 1 (PD-L1) expression, Epstein-Barr virus (EBV) infection status, and heightened tumor mutational burden (TMB) (11, 12). Findings from the DANTE research indicated that patients with higher combined positive scores (CPS) achieved greater improvements in pCR, suggesting that PD-L1 expression is positively correlated with immunotherapy efficacy (8). Nonetheless, these biomarkers do not appear to consistently predict treatment response. For instance, the KEYNOTE-585 trial reported no improvement in outcomes among PD-L1 positive patients receiving immunotherapy versus those treated solely with chemotherapy (7). Furthermore, several investigations have reported that individuals with microsatellite-stable (MSS) and PD-1/PD-L1 negative gastric carcinoma may still derive therapeutic benefit from ICIs (13). Other studies have shown that ICIs can enhance overall survival (OS) and progression-free survival (PFS) in patients with gastric cancer, irrespective of PD-L1 expression levels (12).

Therefore, accurately identifying patients most suited for immunotherapy is essential. In the research, we further examined NICT related biomarkers in patients with LAGC and developed a predictive model for pathological treatment response in this population. This model may assist clinicians in precisely selecting candidates most likely to benefit and in formulating more effective treatment strategies, thereby potentially improving survival outcomes.

## 2 Materials and methods

### 2.1 Research subjects

This retrospective analysis enrolled 113 patients with gastric cancer who were treated with a combination of neoadjuvant chemotherapy and immunotherapy at the Affiliated Hospital of North Sichuan Medical College between January 2021 and March 2025. All patients subsequently underwent radical gastrectomy. Criteria for inclusion included the following: (1) Clinical staging of cT2N+M0 or cT3–4bN0–3M0 gastric cancer; (2) Preoperative pathological confirmation of a primary gastric lesion, without evidence of metastatic disease; (3) Minimum two sequential rounds of neoadjuvant chemotherapy integrated with immunotherapy prior to radical gastrectomy; (4) Availability of detailed clinicopathological information. Criteria for exclusion included the following: (1) Presence of malignant tumors at other sites; (2) Receipt of any additional antitumor therapy before surgery, including radiotherapy or targeted therapy; (3) History or current diagnosis of autoimmune disease or serious organic disorders. This retrospective study was approved by the Ethics Committee of North Sichuan Medical College’s Affiliated Hospital (Approval No. 2024ER492-1). Written informed consent was waived.

### 2.2 Data collection

All included patients received NICT, consisting of a chemotherapy backbone combined with a PD-1 inhibitor. Chemotherapy regimens comprised SOX (S-1 + oxaliplatin), DOS (docetaxel + oxaliplatin + S-1), FLOT (fluorouracil + oxaliplatin + docetaxel), FOLFOX (oxaliplatin + fluorouracil), and XELOX (capecitabine + oxaliplatin). The PD-1 inhibitor administered included sintilimab, tislelizumab, camrelizumab, pembrolizumab and nivolumab. Patients received at least two consecutive cycles of NICT. The treatment-related adverse events (TRAEs) were categorized and graded according to the Common Terminology Criteria for Adverse Events criteria (version 5.0) during NICT1 (14).

We gathered and examined initial clinical and pathological information for individuals diagnosed with LAGC, such as their age, gender, height, weight, body mass index (BMI), smoking status, alcohol consumption, clinical stage, tumor bed diameter, tumor location, histological differentiation, Lauren classification, Borrmann classification, postoperative pathological T stage (ypT), postoperative pathological N stage (ypN), vascular invasion, perineural invasion, and tumor regression grade (TRG). Peripheral blood tests were performed prior to the first cycle of NICT. Laboratory parameters included white blood cell count, hemoglobin, lymphocyte count, neutrophil count, platelet count, tumor markers, albumin, total cholesterol, and lactate dehydrogenase levels. Abdominal computed tomography (CT) scans with contrast were administered before and after NICT to evaluate treatment efficacy.

### 2.3 Efficacy evaluation

In this study, major pathological response (MPR) to neoadjuvant immunochemotherapy (NICT) in LAGC patients was used as the primary outcome measure. MPR was characterized as having no more than 10% viable tumor cells in the primary tumor site, regardless of metastatic lymph node involvement. Postoperative pathological tumor regression was graded based on the Becker criteria (15): TRG 1a (complete absence of residual tumor cells), TRG 1b (residual tumor cells occupying *<* 10% of the tumor bed), TRG 2 (tumor cellularity between 10% and 50%), TRG 3 (residual tumor cells exceeding 50%). Patients were classified into two categories based on their pathological reaction to NICT: the MPR cohort (TRG 1a and 1b) and the non-MPR cohort (TRG 2 and 3). The MPR group demonstrated tumor regression corresponding to TRG 1a or 1b, indicating a good therapeutic response, whereas the non-MPR group showed poorer pathological outcomes consistent with TRG 2 or TRG 3. Clinical and pathological characteristics were contrasted across the MPR and non-MPR groups, with independent MPR determinants identified.

Radiologic treatment response was assessed according to the Response Evaluation Criteria in Solid Tumors (RECIST) version 1.1 (16). Four categories of response were defined: complete response (CR), partial response (PR), stable disease (SD), and progressive disease (PD). Complete response (CR) was defined as the disappearance of all tumor lesions, with the change maintained for at least four weeks. Partial response (PR) was defined as a reduction of 30% or more in the size of target lesions, with the change maintained for at least four weeks. Stable disease (SD) was marked by either a reduction of under 30% or a growth of less than 20% in target lesions, provided these changes remained consistent for at least four weeks. Progressive disease (PD) was identified when there was a swelling of 20% or more in target lesion diameter, a proportional increase in existing lesions, or the appearance of new lesions.

### 2.4 Calculation of inflammatory indicators

Inflammatory response indicators included the neutrophil/lymphocyte ratio (NLR, neutrophil count/lymphocyte count), the platelet/lymphocyte ratio (PLR, platelet count/lymphocyte count), systemic immune-inflammation index (SII) score [platelet count (×10^9^/L) × neutrophil count (×10^9^/L)/lymphocyte count (×10^9^/L)] (17) and the prognostic nutritional index (PNI) score [serum albumin (g/L) + 5 × lymphocyte count (×10^9^/L)] (18). APRI (Aspartate aminotransferase-to-platelet ratio index) = aspartate aminotransferase level (U/L)/platelet count (×10^9^/L); RPR (Red cell distribution width-to-platelet ratio) = red cell distribution width (%)/platelet count (×10^9^/L); PLPR (Platelet-to-prealbumin-lymphocyte ratio) = platelet count (×10^9^/L)/[serum prealbumin (g/L)× lymphocyte count (×10^9^/L)]; APR (Alkaline phosphatase-to-prealbumin ratio) = alkaline phosphatase level (U/L)/serum prealbumin (g/L); DIR (Direct-to-indirect bilirubin ratio) = direct bilirubin level (*µ*mol/L)/indirect bilirubin level (*µ*mol/L).

### 2.5 Statistical analysis

All statistical computations were carried out with R software (version 4.5.0). Student’s t-test was employed to analyze normally distributed continuous data, reporting findings as mean ± standard deviation (SD). For continuous variables that deviated from normal distribution, comparisons were performed using the Wilcoxon rank-sum test, and results were expressed as the median accompanied by the interquartile range [M (IQR)]. Categorical variables were presented as frequencies with corresponding percentages and compared between groups using the chi-square test, while ordinal variables were compared using the Mann–Whitney U test. The least absolute shrinkage and selection operator (LASSO) regression method was used for feature selection and identification of predictive variables. Receiver operating characteristic (ROC) curves were performed, and the Youden index was calculated to determine optimal cut-off values for pre-treatment tumor bed diameter, carcinoembryonic antigen (CEA), carbohydrate antigen 19-9(CA19-9), neutrophil-to-lymphocyte ratio (NLR), and systemic immune-inflammation index (SII), converting these variables into binary form. Variables selected by LASSO regression were entered into multivariable logistic regression analysis, and those with statistical significance were used to construct a nomogram. Nomogram ROC curves were plotted, with the area under the curve (AUC) values computed to evaluate prediction accuracy. Internal validation employed bootstrap resampling (1,000 iterations), with model calibration evaluated via the Hosmer–Lemeshow goodness-of-fit test. Clinical utility of the nomogram was assessed via decision curve analysis (DCA). A P-value below 0.05 indicated statistical significance.

## 3 Results

### 3.1 Baseline characteristics of MPR and non-MPR cohorts after NICT in LAGC patients

113 patients with LAGC received radical surgery following NICT were enrolled in the research. Among them, 85 (75.22%) were male and 28 (24.78%) were female. Diagnosed at an age range of 55 to 69 years, the average age of the patients was 63. In terms of treatment characteristics, chemotherapy regimens were comparable between groups (P = 0.766). Overall, SOX (49.56%) and DOS (39.82%) were the most frequently used backbones, followed by FOLFOX (5.31%), FLOT (4.42%), and XELOX (0.88%). The PD-1 inhibitors used included sintilimab, tislelizumab, and camrelizumab, and the distribution of immunotherapy regimens (domestic vs imported) did not differ significantly between groups (P = 0.139). The number of neoadjuvant cycles (*<*4 vs ≥4) was also similar between groups (P = 1.000). Based on pathological response assessment of surgically resected specimens, participants were divided into those achieving a MPR and those without MPR (**Table 1**). The MPR group comprised 46 patients who achieved TRG1a/1b, while the non-MPR group comprised 67 patients who achieved TRG2/3, corresponding to an overall MPR rate of 40.7%. No statistically meaningful variations were observed in the initial attributes of the two groups, including sex, age, smoking and drinking habits, tumor location, chemotherapy regimens, immunotherapy regimens, treatment cycles and preoperative imaging stage (all P *>* 0.05). Relative to the non-MPR cohort, patients in the MPR cohort demonstrated better radiologic treatment responses (P = 0.0001), smaller tumor bed diameter (P = 0.0321), and earlier pathological stage (P ≤ 0.001). Regarding tumor biomarkers, the MPR group exhibited markedly reduced CEA and CA19–9 levels compared to the non-MPR group (P = 0.0109 and P = 0.029, respectively). In terms of inflammatory markers, APRI and RPR levels proved markedly elevated in the MPR group (P = 0.0197 and P = 0.0023, respectively), whereas PLPR, PLR, and SII levels were markedly reduced (P = 0.0012, P = 0.0006, and P = 0.0021, correspondingly).

**Table 1:** Baseline characteristics comparison between MPR and non-MPR groups in locally advanced gastric cancer patients before NICT.

Characteristics	All patients (n=113)	MPR (N = 46)	Non-MPR (N = 67)	P
Gender (%)				
Female	28 (24.78)	11 (23.91)	17 (25.37)	1
Male	85 (75.22)	35 (76.09)	50 (74.63)	
Age (median [IQR])	63.000 [55.000, 69.000]	59.000 [55.000, 70.000]	65.000 [55.500, 68.500]	0.8953
BMI (median [IQR])	21.641 [19.922, 24.024]	22.039 [21.094, 25.391]	21.484 [19.547, 23.340]	0.0414
Tobacco (%)				
No	62 (54.87)	28 (60.87)	34 (50.75)	0.3843
Yes	51 (45.13)	18 (39.13)	33 (49.25)	
Alcohol (%)				
No	72 (63.72)	32 (69.57)	40 (59.70)	0.3831
Yes	41 (36.28)	14 (30.43)	27 (40.30)	
Tumor Location (%)				
Cardia fundus	53 (46.90)	18 (39.13)	35 (52.24)	0.282
Body of stomach	29 (25.66)	12 (26.09)	17 (25.37)	
Antrum of stomach	31 (27.43)	16 (34.78)	15 (22.39)	
Chemotherapy regimens (%)				
SOX	56 (49.56)	33 (49.25)	23 (50.00)	0.766
DOS	45 (39.82)	28 (41.79)	17 (36.96)	
FLOT	5 (4.42)	2 (2.99)	3 (6.52)	
FOLFOX	6 (5.31)	3 (4.48)	3 (6.52)	
XELOX	1 (0.88)	1 (1.49)	0 (0.00)	
Immunotherapy regimens (%)				
Domestic drug	95 (84.07)	53 (79.10)	42 (91.30)	0.139
Import drug	18 (15.93)	14 (20.90)	4 (8.70)	
Treatment cycles (%)				
*<*4	61 (53.98)	36 (53.73)	25 (54.35)	1.000
≥4	52 (46.02)	31 (46.27)	21 (45.65)	
T (%)				
2	1 (0.88)	0 (0.00)	1 (1.49)	0.5379
3	66 (58.41)	29 (63.04)	37 (55.22)	
4	46 (40.71)	17 (36.96)	29 (43.28)	
N (%)				
0	1 (0.88)	1 (2.17)	0 (0.00)	0.5522
1	18 (15.93)	6 (13.04)	12 (17.91)	
2	70 (61.95)	30 (65.22)	40 (59.70)	
3	24 (21.24)	9 (19.57)	15 (22.39)	
Radiological response (%)				
CR	11 (9.73)	11 (23.91)	0 (0.00)	0.0001
PR	45 (39.82)	18 (39.13)	27 (40.30)	
SD	53 (46.90)	17 (36.96)	36 (53.73)	
PD	4 (3.54)	0 (0.00)	4 (5.97)	
ypT (%)				
T0-T2	34 (30.09)	25 (54.35)	9 (13.43)	*<*0.0001
T3-T4	79 (69.91)	21 (45.65)	58 (86.57)	
ypN (%)				
N0	40 (35.40)	25 (54.35)	15 (22.39)	0.001
N1-N3	73 (64.60)	21 (45.65)	52 (77.61)	
Tumor bed diameter (median [IQR])	3.500 [3.000, 5.000]	3.050 [2.500, 4.000]	4.000 [3.000, 5.000]	0.0321
Nervous invasion (%)				
No	81 (71.68)	37 (80.43)	44 (65.67)	0.1339
Yes	32 (28.32)	9 (19.57)	23 (34.33)	
Borrmann (%)				
I Type	3 (2.65)	0 (0.00)	3 (4.48)	0.0003
II Type	32 (28.32)	23 (50.00)	9 (13.43)	
III Type	75 (66.37)	22 (47.83)	53 (79.10)	
IV Type	3 (2.65)	1 (2.17)	2 (2.99)	
Lauren (%)				
Intestinal pattern	79 (69.91)	37 (80.43)	42 (62.69)	0.0997
Diffuse type	26 (23.01)	6 (13.04)	20 (29.85)	
Mixed type	8 (7.08)	3 (6.52)	5 (7.46)	
Differentiation (%)				
G3-G4	29 (25.66)	12 (26.09)	17 (25.37)	1
G1-G2	84 (74.34)	34 (73.91)	50 (74.63)	
Leucocyte (median [IQR])	5.920 [4.910, 7.340]	5.885 [4.822, 7.220]	5.960 [5.100, 7.480]	0.5807
Neutrophil (median [IQR])	4.080 [3.200, 5.530]	3.855 [3.180, 5.422]	4.360 [3.295, 5.630]	0.3219
Lymphocyte (median [IQR])	1.280 [0.930, 1.550]	1.455 [0.923, 1.720]	1.190 [0.995, 1.410]	0.0162
Monocyte (median [IQR])	0.350 [0.280, 0.450]	0.330 [0.283, 0.448]	0.360 [0.285, 0.445]	0.8561
Platelet (median [IQR])	247.000 [194.000, 285.000]	213.000 [182.000, 258.000]	259.000 [211.500, 298.000]	0.0032
Albumin (mean (SD))	41.132 (4.151)	41.350 (4.586)	40.981 (3.853)	0.645
CEA (median [IQR])	2.240 [0.870, 11.280]	1.520 [0.675, 3.930]	2.900 [1.080, 24.020]	0.0109
CA19-9 (median [IQR])	18.380 [9.600, 41.090]	13.800 [9.375, 25.083]	23.510 [12.440, 48.100]	0.0297
DIR (median [IQR])	0.521 [0.444, 0.627]	0.515 [0.471, 0.577]	0.523 [0.444, 0.661]	0.5728
APRI (median [IQR])	0.080 [0.060, 0.126]	0.096 [0.066, 0.154]	0.074 [0.058, 0.103]	0.0197
RPR (median [IQR])	0.060 [0.048, 0.073]	0.067 [0.056, 0.084]	0.056 [0.047, 0.066]	0.0023
PLPR (median [IQR])	1.076 [0.728, 1.486]	0.796 [0.639, 1.324]	1.196 [0.869, 1.761]	0.0012
APR (median [IQR])	0.480 [0.360, 0.599]	0.483 [0.404, 0.630]	0.479 [0.359, 0.578]	0.4941
NLR (median [IQR])	3.232 [2.366, 4.843]	2.796 [2.205, 4.334]	3.536 [2.512, 5.208]	0.0344
PLR (median [IQR])	195.556 [140.000, 270.909]	151.221 [111.754, 212.160]	217.518 [169.238, 281.146]	0.0006
SII (median [IQR])	756.800 [539.783, 1238.524	600.508 [432.944, 989.606]	900.249 [650.195, 1349.696]	0.0021
SIRI (median [IQR])	1.115 [0.803, 1.790]	1.045 [0.686, 1.605]	1.142 [0.851, 2.135]	0.2425
PNI (median [IQR])	48.300 [44.400, 50.250]	49.175 [45.137, 51.112]	47.900 [43.975, 49.825]	0.1301

### 3.2 Safety and adverse events

Overall, adverse events (AEs) were common but largely manageable. Grade 1or 2 adverse events were common, occurring in 96 patients (85.0%). The most frequent TRAEs were anemia (66.4%), leukopenia (46.9%), nausea or vomiting (45.1%), decreased appetite (44.2%), elevated ALT/AST (29.2%), and diarrhea (13.7%). Grade 3 adverse events were less frequent and occurred in 20 patients (17.7%). The most common grade 3 toxicities included anemia (4.4%), thrombocytopenia (3.5%), elevated ALT/AST (3.5%), diarrhea (3.5%), and leukopenia (2.7%). Immune-associated adverse events (irAEs) were mainly hypothyroidism (all grade 13.3%) and rash (1.8%) (**Supplementary Table 2**).

### 3.3 Predictors of MPR

**Figure 1:**
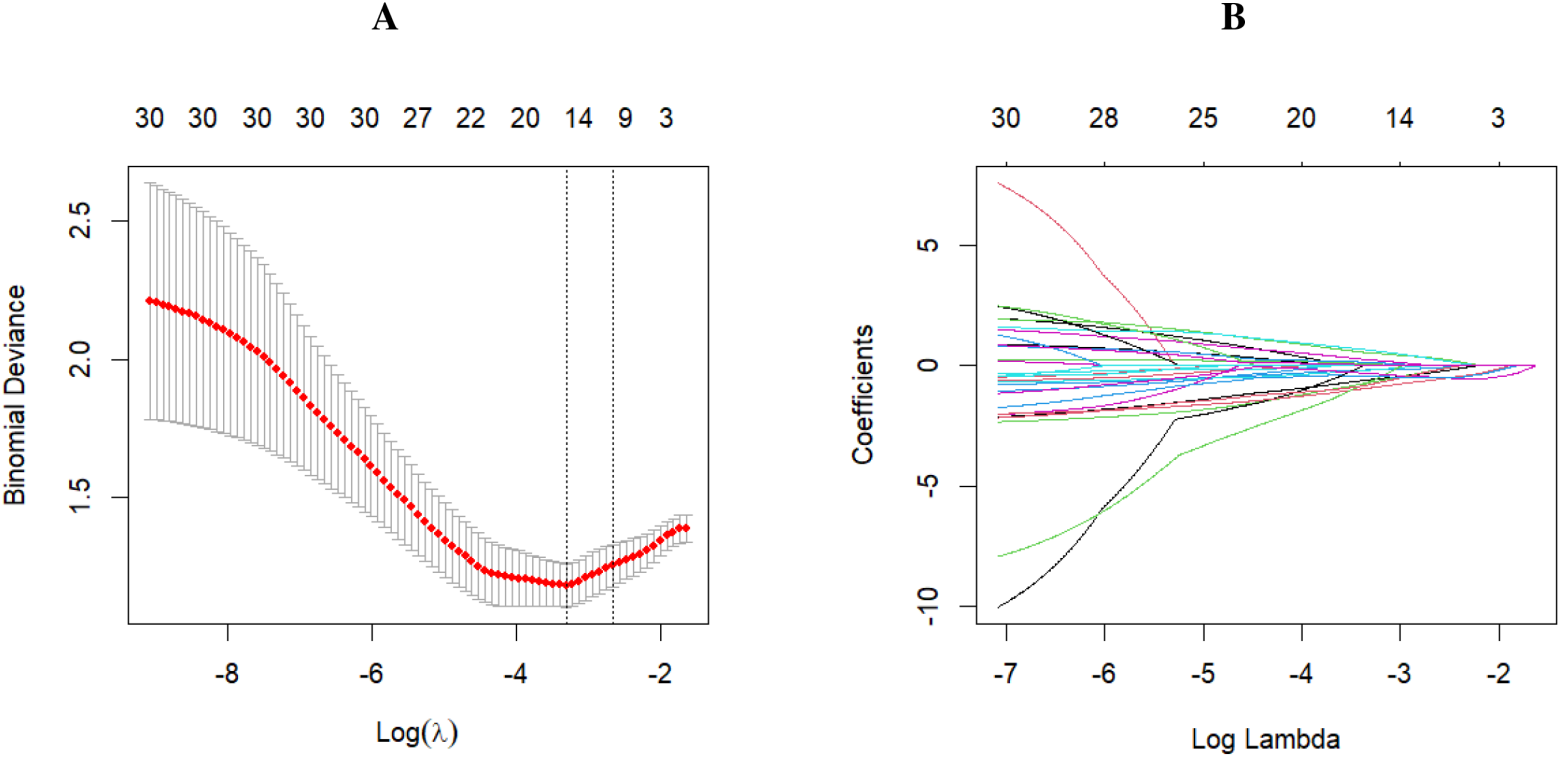
The selection of predictors was conducted utilizing LASSO regression analysis accompanied by tenfold cross-validation. **(A)** The analysis included selection of the tuning parameter (*λ*), with model deviance in LASSO regression assessed using two criteria: the minimum criterion (marked by the left dotted line) and the 1-SE criterion (marked by the right dotted line). **(B)** The coefficient profile was plotted against the logarithmic sequence of *λ* values. LASSO, least absolute shrinkage and selection operator; SE, standard error.

Receiver operating characteristic (ROC) curves were plotted, with optimal threshold of each variable determined by the Youden index. LASSO regression analysis indicated that predictors were chosen according to the 1-SE criterion, yielding six nonzero coefficients. They include tumor bed diameter, CEA, CA19-9, NLR, SII, and PNI (**Figure 1**). These factors were subsequently incorporated into a multivariate logistic regression to develop a predictive model for pathological response (**Table 2**). The findings revealed that tumor bed diameter *<* 3.75 cm (odds ratio [OR] [95% confidence interval (CI)]: 0.22[0.072 0.601], P = 0.00481), CEA *<* 1.765 ng/mL [OR (95% CI): 0.26 (0.088–0.70), P = 0.00984], CA19-9 *<* 18.390 U/mL [OR (95% CI): 0.148 (0.046–0.415), P *<* 0.001], NLR *<* 2.422 [OR (95% CI): 0.265 (0.068–0.971), P = 0.04746], and SII *<* 597.483 [OR (95% CI): 0.194 (0.052–0.645), P = 0.00992] were independent predictors of achieving MPR after NICT in patients with LAGC.

**Table 2:** Multivariate logistic regression analysis of predictors of major pathological response (MPR) following NICT in patients with LAGC.

Variable	B	SE	Wald	OR with CI	P
(Intercept)	3.081	1.179	6.826	21.785 (2.492–263.329)	0.009
Tumor bed diameter	-1.393	0.549	6.443	0.248 (0.079–0.697)	0.011
CEA	-1.321	0.527	6.286	0.267 (0.090–0.727)	0.012
CA19-9	-1.897	0.557	11.600	0.150 (0.047–0.423)	*<*0.001
NLR	-1.330	0.664	4.010	0.265 (0.068–0.957)	0.045
SII	-1.558	0.637	5.984	0.210 (0.056–0.702)	0.014
PNI	0.512	0.505	1.029	1.669 (0.617–4.541)	0.310

### 3.4 Development and internal validation of a nomogram for MPR prediction

**Figure 2:**
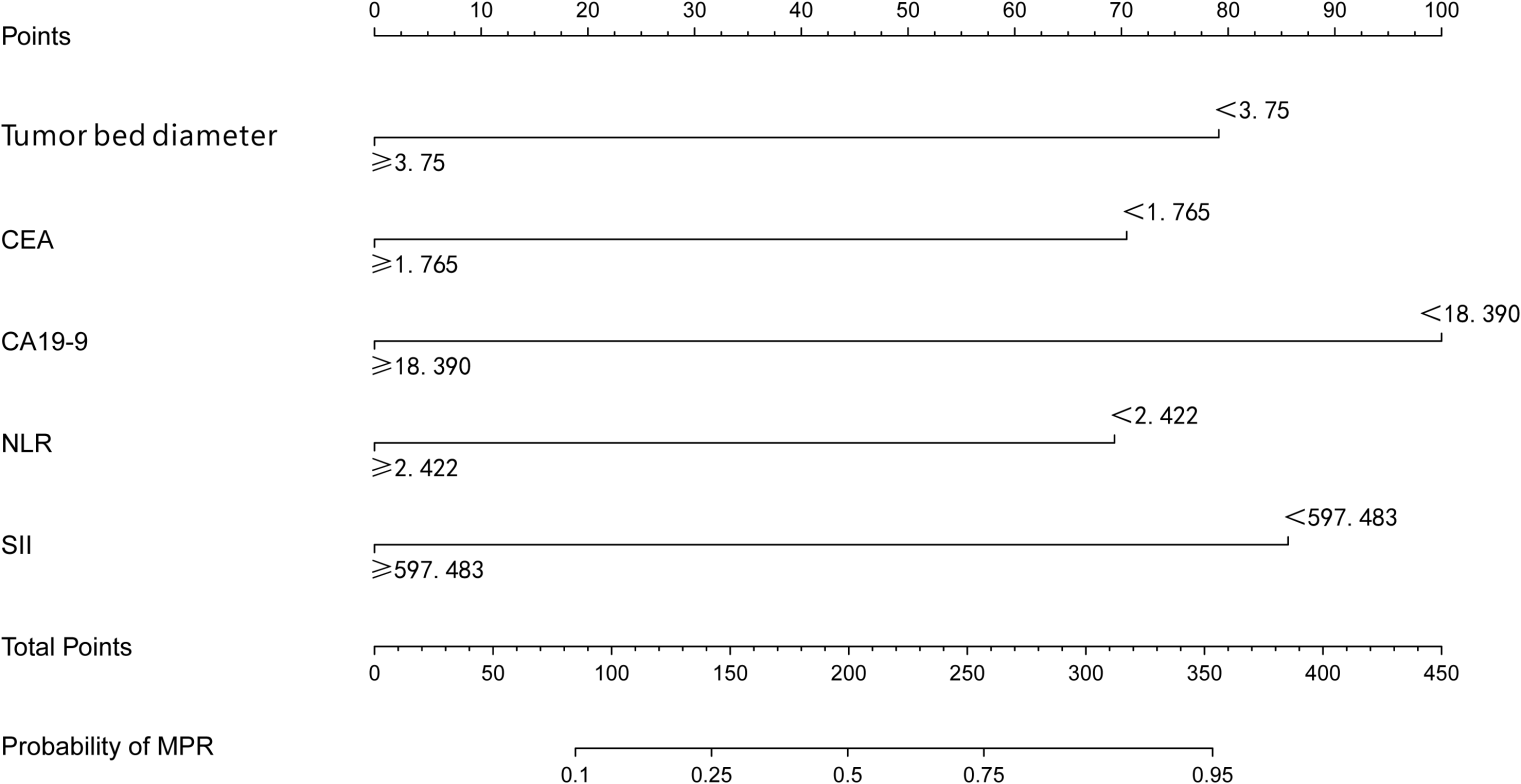
The developed nomogram estimates the likelihood of achieving a MPR following NICT in individuals diagnosed with LAGC. On the vertical axis, various factors influencing MPR are illustrated, which include tumor bed diameter, CEA, CA19-9, NLR, and SII. The top horizontal scale represents the point allocation for each predictor, with values varying between 0 and 100. In contrast, the bottom horizontal scale indicates the total accumulated score, extending from 0 to 450, together with the associated estimated risk, which ranges from 0.10 to 0.95. MPR, major pathological response; NICT, neoadjuvant immunochemotherapy; LAGC, locally advanced gastric cancer.

**Figure 3:**
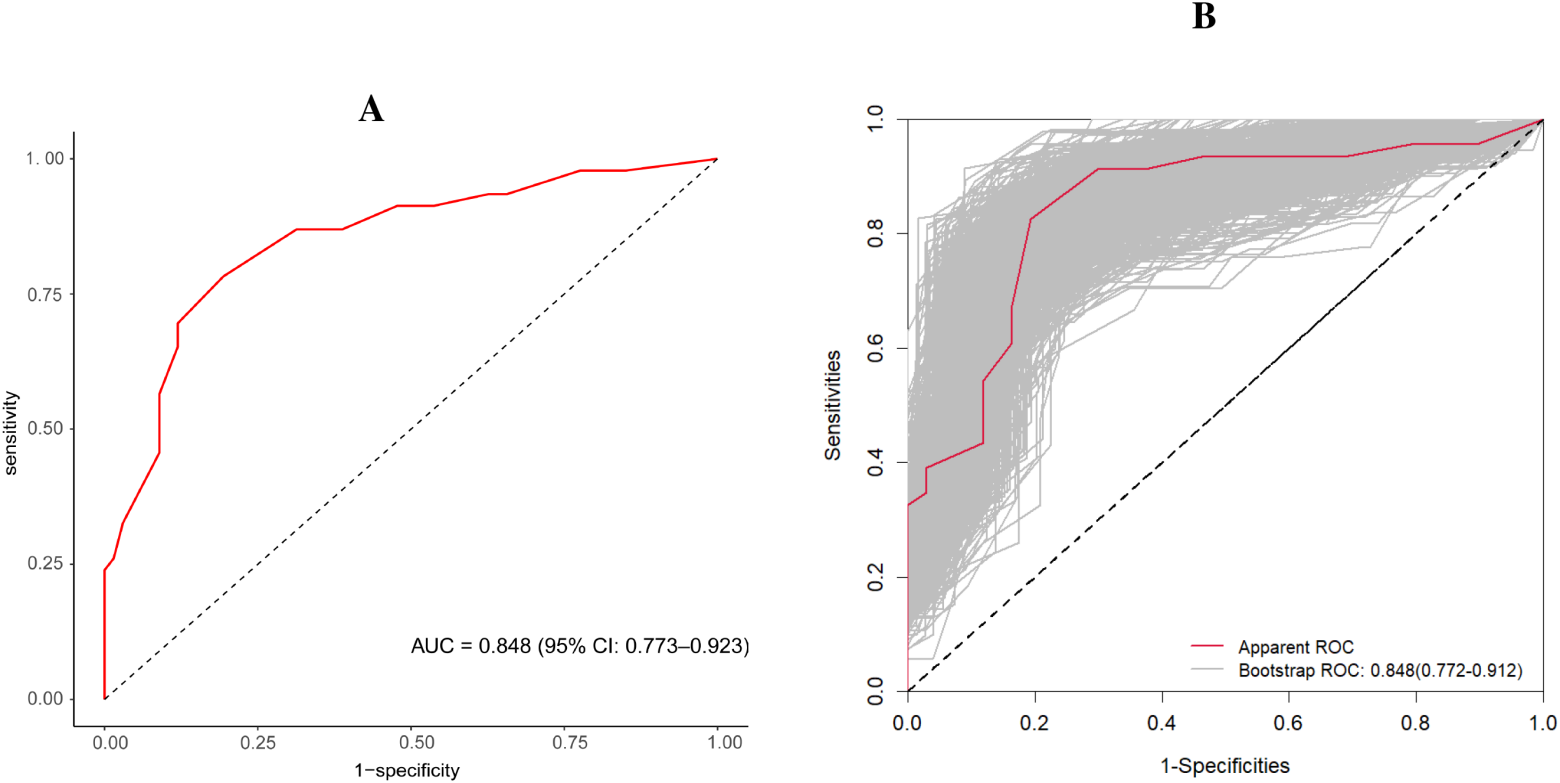
The AUC of the model and the internal validation. **(A)** illustrates the AUC of the predictive model, and **(B)** shows the AUC of the internal validation using the bootstrap method (resampling = 1000). AUC, area under the curve.

**Figure 4:**
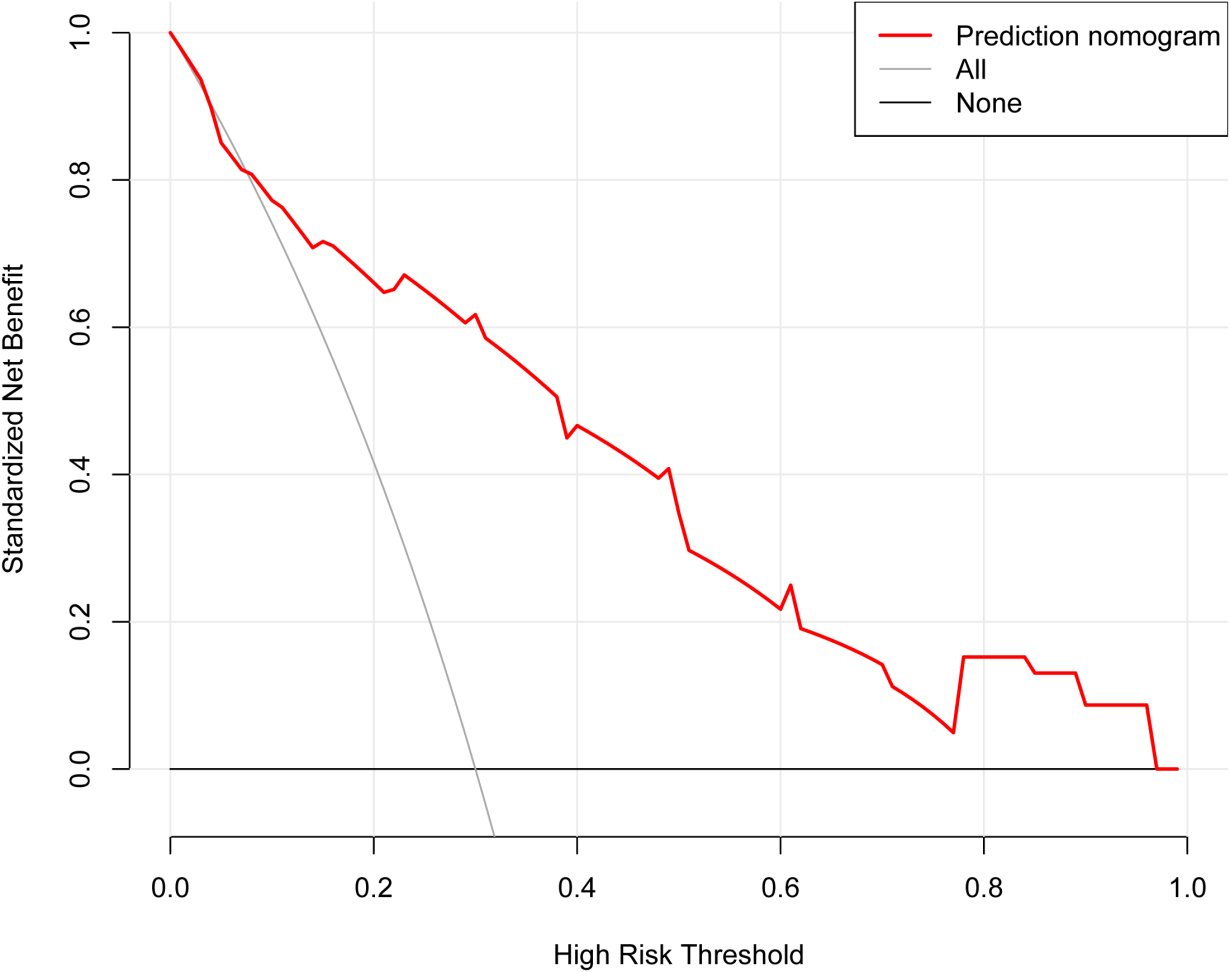
The decision curve analysis of the nomogram. On the horizontal scale, threshold probabilities are plotted, whereas the vertical scale represents the corresponding net benefit values. Over the continuous range of threshold probabilities, the net benefit trajectory for the prediction model is shown by the red line. The gray curve illustrates the net benefit if all cases were classified as positive, and the black curve shows the net benefit if all cases were classified as negative. This visualization system illustrates the clinical utility and economic value of the prediction model when weighing varying costs and benefits of decision-making.

**Figure 5:**
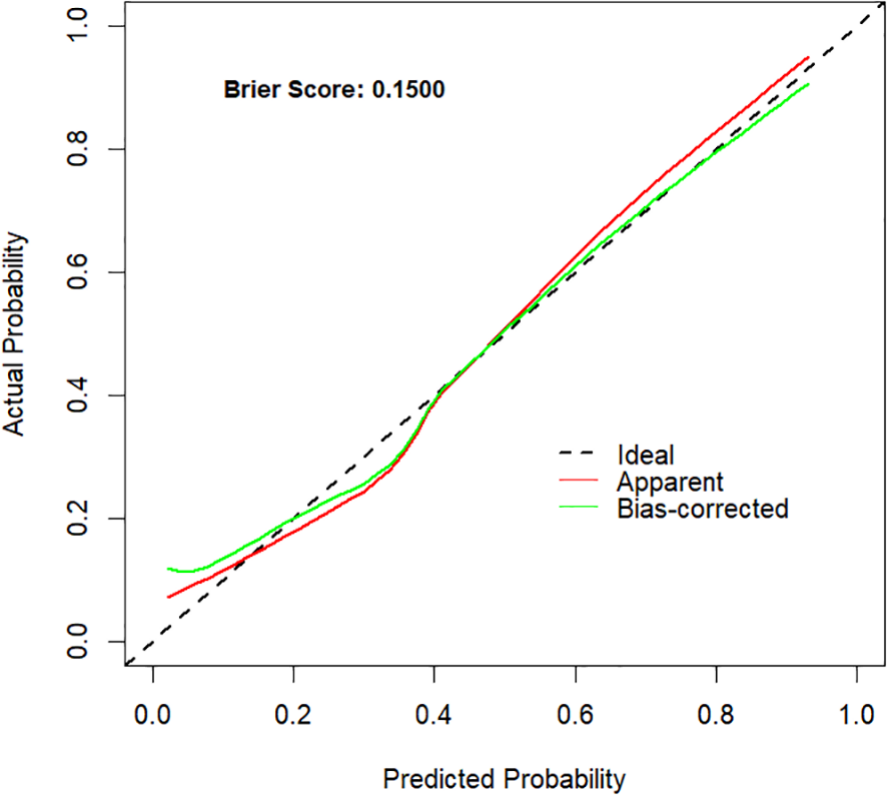
The calibration curve of the nomogram. The x-axis displays the model’s predicted probabilities, whereas the y-axis depicts the corresponding observed outcomes. The Brier score was 0.15. The red curve represents the apparent calibration before any bias adjustment, whereas the green curve reflects the bias-corrected calibration, showing improved alignment with the ideal reference line following correction.

A nomogram model was constructed to predict the likelihood of achieving MPR after NICT in LAGC patients, incorporating tumor bed diameter, gastric tumor markers (CEA and CA19-9), and inflammatory indices (NLR and SII) (**Figure 2**). The ROC curve derived from the nomogram showed an AUC of 0.848 (95% CI: 0.773–0.923). Internal validation was performed using the bootstrap method (resampling times=1,000), yielding an AUC value of 0.848 (95% CI: 0.772–0.912) (**Figure 3**). The proposed model demonstrated good calibration (**Figure 4**), with a *χ*^2^ goodness-of-fit value by Hosmer-Lemeshow of 4.705 and a non-significant P value of 0.789, showing no statistically significant discrepancy in the comparison of predicted and observed probabilities. The Brier score was 0.15, suggesting a relatively low overall prediction error. To further evaluate its clinical utility, decision curve analysis (DCA) was employed. The DCA indicated that the nomogram provided a moderate additional net benefit in predicting MPR after NICT in LAGC patients, suggesting potential applicability in clinical decision (**Figure 5**).

To further clarify the applicable scope of the model, we performed subgroup analyses across NICT regimens and tumor subtypes (**Supplementary Figure 1**). Due to the limited sample size, chemotherapy regimens were categorized into two groups, doublet regimens and triplet regimens. The AUCs were broadly comparable across levels of chemotherapy regimens, immunotherapy regimens, treatment cycles, Borrmann type, Lauren classification, and differentiation. No statistically significant differences in AUC were observed between levels within each subgroup variable (all P values *>* 0.05). Moreover, interaction testing did not identify significant heterogeneity (all P for interaction *>* 0.05), suggesting that the discrimination of the model was generally consistent across these strata.

## 4 Discussion

With the widespread ICIs in gastric cancer, neoadjuvant immunochemotherapy has attracted increasing attention due to its superior anti-tumor efficacy compared with neoadjuvant chemotherapy alone, and has gradually become the main neoadjuvant therapy option for LAGC patients. Nevertheless, reliable clinical biomarkers to predict pathological response to first-line NICT are currently lacking. Therefore, the development of a predictive model for tumor regression is of great importance, as it can help clinicians identify immunotherapy sensitive populations.

Several nomograms have been proposed to predict pathological response to neoadjuvant therapy in LAGC, yet the selected predictors, validation strategies, and clinical utility assessments vary across studies. Li et al. developed an internally validated nomogram for predicting pCR in LAGC patients treated with NICT. Their final model incorporated clinical N stage, CPS, and tumor diameter, achieving a C-index of 0.923 with bootstrap internal validation and calibration assessment, although a decision curve based evaluation of clinical benefit was not reported (19). More recently, Cui et al. reported a multicenter real-world prediction model for pCR after NICT. The nomogram demonstrated strong discrimination with AUCs of 0.862 and 0.934 in the training and validation cohorts, respectively, and further showed good calibration (Hosmer–Lemeshow P ¿ 0.05) and favorable DCA (20). In addition, Shao et al. developed a nomogram to predict MPR after neoadjuvant therapy. The model included pretreatment differentiation, clinical T stage, monocyte count, CA724, and receipt of neoadjuvant chemoradiotherapy (nCRT), and achieved an AUC of 0.777 (21). Rather than using tumor-tissue biomarkers such as PD-L1 CPS or molecular testing, we built a parsimonious nomogram based on routinely available, low-cost variables, including tumor bed diameter, CEA, CA19-9, NLR, and SII. This design improves real-world applicability, especially in settings where biomarker testing is incomplete. The model was internally validated using bootstrap resampling (1,000 iterations). It showed stable discrimination (AUC 0.848) and good calibration (Hosmer–Lemeshow test: *χ*^2^ = 4.705, *P* = 0.789). Decision curve analysis further suggested potential clinical utility. This tool may assist clinicians in recognizing immunotherapy-sensitive 243 patients and in selecting the optimal timing for surgery.

At present, a substantial number of investigations have explored the use of NICT in individuals with LAGC prior to surgery. Preliminary data from several trials based on PD-1 inhibitor containing neoadjuvant regimens have been reported. In a phase II trial with a single-armed design, neoadjuvant therapy of sintilimab together with oxaliplatin and capecitabine for resectable locally advanced G/GEJ adenocarcinoma achieved a MPR rate of 47.2% (22). Another study demonstrated that the neoadjuvant regimen incorporating camrelizumab alongside nab-paclitaxel and S-1 achieved a pCR rate of 39.4% in patients with serosa-invasive gastric cancer (23). In a separate phase 2 trial, SOX chemotherapy with tislelizumab as neoadjuvant therapy resulted in a 53.1% rate of MPR in locally advanced resectable G/GEJ cancer patients (24). In addition, Sun et al. compared patients receiving NICT with those receiving NCT using propensity score matching. Their findings showed that, compared to the NCT group, the NICT cohort indicated a superior objective response (79.5% vs. 59.0%) and a reduced early recurrence rate (29.7% vs. 40.8%) (25). In our study, the MPR rate in LAGC patients following NICT was 40.7%. This finding reflects the promising therapeutic performance of NICT and suggests potential long-term survival for the patient cohort.

CEA and CA19-9, predominantly produced by abnormal tumor tissues, are both tumor-associated glycoproteins widely recognized as markers of gastrointestinal cancers. They are commonly used in tumor diagnosis, recurrence monitoring, and evaluation of therapeutic efficacy. However, their potential value in predicting the efficacy of NICT remains incompletely defined (26). An analysis of 399 LAGC patients who receiving NACT followed by surgery reported that 33.1% of them had higher pre-NACT CEA and/or CA19–9 levels. Moreover, CEA and CA19–9 levels before and after neoadjuvant chemotherapy showed a substantial association with patient prognosis (p = 0.0023) (27). Another study incorporated CA72–4 into the analysis and developed a composite scoring system of post-NACT tumor markers (post-NACT CTM score) comprising CEA, CA19-9, and CA72-4. Higher pCR rates and longer progression-free survival (PFS) were observed in those with lower CTM scores (0–1). Specifically, compared to scores of 2/3, patients with a CTM score of 0 was associated with an odds ratio of 4.33 for achieving pCR (P = 0.012). Multivariate analysis established CTM scoring as a standalone indicator of PFS (28). It is generally believed that overexpressed CEA proteins accumulate on the cell membrane, interfering with normal growth inhibition and cell differentiation, thereby promoting tumor progression (29). CA19–9 is a high molecular weight mucin antigen that is believed to act as an opposing adhesion molecule, facilitating carcinoma metastasis while regionally inhibiting antitumor immunity mediated by T cells (30). In the current investigation, we discovered that individuals with LAGC who had preoperative CEA levels below 1.765 ng/mL and CA19–9 levels below 18.390 U/mL were more likely to achieve a MPR than those presenting with higher biomarker levels. These findings demonstrate that lower preoperative CEA and CA19–9 levels may serve as promising biomarkers in predicting enhanced tumor regression following NICT. Consequently, attention should be paid to preoperative CEA and CA19–9 concentrations when assessing the optimal timing for surgery during the course of NICT.

Inflammatory processes are integral to the tumor microenvironment and have strong associations with the onset, advancement, and metastatic spread of malignancies. An increasing amount of research suggests that the pathogenesis of various solid malignant tumors is significantly influenced by systemic inflammatory mediators (31). Key participants in the systemic inflammatory process induced by tumors include a variety of inflammatory cells, such as neutrophils, lymphocytes, monocytes, and platelets (32, 33). This response may facilitate tumor development and dissemination via multiple mechanisms, including enhanced secretion of inflammatory mediators and cytokines, suppressing apoptosis, and inducing DNA damage in tumor cells (34). Several biomarkers have been utilized to assess prognosis in numerous malignancies, including NLR, the lymphocyte-to-monocyte ratio (LMR), the platelet-to-lymphocyte ratio (PLR), and SII (35, 36). These indices capture patterns of immune and inflammatory cell balance that can reflect host–tumor interactions, and their clinical utility has been increasingly recognized in prognostic modeling.

The ratio of neutrophils to lymphocytes is a blood-based circulating biomarker, while the SII is calculated as the neutrophil count multiplied by the platelet count, divided by the lymphocyte count. Both indicators have been used to estimate the therapeutic effectiveness of ICIs therapy across multiple cancer types, and have attracted considerable attention due to their low cost and wide availability (37). Prior research indicates that certain preoperative hematological markers, including NLR and PLR, play a prognostic role after surgery in gastric cancer patients. Specifically, the increased NLR is strongly linked to significantly reduced OS and PFS (38). Sustained elevation of NLR from the preoperative to postoperative period has also been demonstrated to a poor predictor of OS in gastric cancer individuals (39). Moreover, according to the analysis by Martin and colleagues, both SII and PD-L1 CPS independently predicted treatment response to nivolumab plus chemotherapy in patients with metastatic gastric adenocarcinoma who had a PD-L1 CPS of ≥ 5 (40). Wu et al. reported, based on an analysis of 521 gastric adenocarcinoma cases, that low pretreatment levels of SII and NLR independently predicted pCR (41). Similarly, Xu et al. demonstrated that increased preoperative peripheral blood SII was associated with worse prognosis (42). Taken together, these results demonstrate the possibility of NLR and SII as readily available and economical biomarkers for predicting therapeutic response and the future outcome in gastric cancer. These results align with our findings. In the current research, multivariate logistic regression analysis identified preoperative NLR below 2.422 and SII below 597.483 as independent predictors of achieving MPR after NICT in patients with LAGC [OR (95% CI): 0.265 (0.068–0.971), p = 0.04746; OR (95% CI): 0.194 (0.052–0.645), p = 0.00992]. Because NLR is determined by neutrophil and lymphocyte counts, and SII is calculated from neutrophil, lymphocyte, and platelet counts. These indices may influence patient outcomes through several biological mechanisms. Initially, neutrophils linked to tumors could be pivotal in the advancement of cancer, as they release substances that control the extracellular matrix and trigger an inflammatory reaction within the tumor microenvironment. Furthermore, increased number or enhanced activity of neutrophils in the tumor microenvironment usually fosters an immunosuppressive milieu, which in turn results in adverse clinical outcomes (43). Lymphocytes also play a pivotal role. Higher peripheral blood lymphocyte counts have been shown to be significantly associated with MPR after NICT, while a reduction in lymphocytes either preoperatively or after treatment leads to impaired cell-mediated immune responses and diminished cytotoxic function, creating a permissive environment for tumor cell growth (44). In addition, platelets are believed to shield cancer cells from immune surveillance and facilitate metastasis (45). We speculate that lower levels of inflammatory markers following treatment reflect a reduced systemic inflammatory state, indicating effective tumor elimination and a higher probability of attaining MPR. Thus, NLR and SII may serve as potential indicators of tumor recurrence and metastasis, and could provide clinicians with early and appropriate guidance for selecting optimal individualized therapeutic strategies.

This investigation has certain limitations. At first, it employed a retrospective, single-center design, which would have caused bias in participant selection. Second, classic immunotherapy biomarkers such as MSI status, PD-L1 expression, and EBV status were not included because these tests were not routinely performed for all patients in our real-world cohort, leading to substantial missing data. In addition, due to the limited sample, internal validation in the research was performed using bootstrap resampling. Future prospective, multicenter studies with independent external cohorts are warranted to externally validate the model, incorporate molecular biomarkers, and further refine its clinical applicability.

## 5 Conclusion

This study demonstrated that NICT achieved a high MPR rate in patients with LAGC. We further identified five independent predictive factors, including tumor bed diameter, CEA, CA19-9, NLR, and SII. Based on these indicators, a nomogram prediction model with favorable discriminative and calibration abilities was constructed. This tool could estimate the treatment effect of NICT in LAGC patients and provide valuable reference for clinicians in identifying populations with an immunological advantage.

## Data Availability

The original contributions presented in the study are included in the article/**Supplementary Material**. Further inquiries can be directed to the corresponding author.
