# Supplementary material for: Development and analysis of a nomogram for predicting pathological response to neoadjuvant immunochemotherapy in locally advanced gastric cancer
Source: Front Oncol. 2026 Mar 10;16:1737833. doi: 10.3389/fonc.2026.1737833 (PMC13008680; doi:10.3389/fonc.2026.1737833)
Supplement: Supplementary file 1 [file DataSheet1.pdf]

Supplementary Material

1 Supplementary Figures and Tables

1.1 Supplementary Table 1. A sensitivity analysis excluding PNI

variable	B	SE	Wald	OR_with_CI	P
(Intercept)	3.923	0.8843	19.683	50.552(10.352~339.845)	< 0.001
Tumor bed diameter	-1.514	0.537	7.949	0.22(0.072~0.601)	0.005
CEA	-1.349	0.523	6.663	0.26(0.088~0.7)	0.010
CA19-9	-1.913	0.555	11.869	0.148(0.046~0.415)	< 0.001
NLR	-1.327	0.669	3.929	0.265(0.068~0.971)	0.047
SII	-1.638	0.635	6.649	0.194(0.052~0.645)	0.010

1.2 Supplementary Table 2. Treatment-related adverse events during NICT

	Grade 1 and 2	Grade 3
All events	96(85.0%)	20(17.7%)
Anemia	75(66.4%)	5(4.4%)
Leukopenia	53(46.9%)	3(2.7%)
Nausea or vomiting	51(45.1%)	3(2.7%)
Decreased appetite	50(44.2%)	2(1.8%)
Elevated ALT/AST	33(29.2%)	4(3.5%)
Platelet count decrease	24(21.2%)	5(4.4%)
Diarrhea	15(13.7%)	4(3.5%)
Hypothyroidism	12(10.6%)	3(2.7%)
Rash	2(1.8%)	0
Pneumonia	2(1.8%)	0

1.3 Supplementary Figure 1. Subgroup analysis for nomogram based on NICT regimens or tumor subtypes

Variable	Count	Percent	OR (95% CI)	P value	P for interaction
**Chemotherapy regimens**					**0.271**
Doublet	63	55.75	4.72 (2.04–10.94)	<0.001	
Triplet	50	44.25	11.18 (2.89–43.17)	<0.001	
**Immunotherapy regimens**					**0.843**
Domestic	95	84.07	6.51 (2.97–14.24)	<0.001	
Imported	18	15.93	5.17 (0.64–41.60)	0.122	
**Treatment cycles**					**0.756**
<4	61	53.98	5.73 (2.13–15.40)	<0.001	
>=4	52	46.02	7.22 (2.48–21.04)	<0.001	
**Borrmann**					**0.562**
I	3	2.65	NA		
II	32	28.32	9.56 (1.77–51.53)	0.009	
III	75	66.37	4.49 (1.89–10.69)	<0.001	
IV	3	2.65	NA		
**Lauren**					**0.045**
Intestinal	79	69.91	5.10 (2.38–10.90)	<0.001	
Diffuse	26	23.01	12.57 (1.45–108.60)	0.021	
Mixed	8	7.08	NA		
**Differentiation**					**0.607**
Well/Moderate	29	25.66	5.18 (1.62–16.52)	0.005	
Poor	84	74.34	7.70 (3.04–19.53)	<0.001	

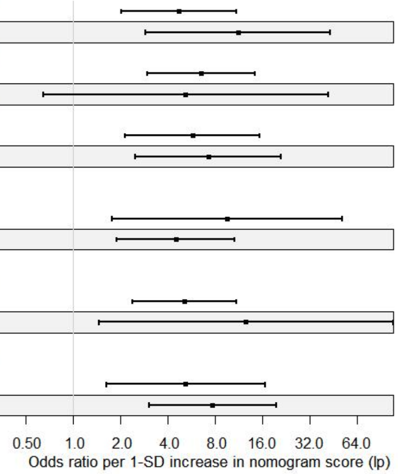

1.4 Supplementary Figure 2. ROC curve of the model including PNI

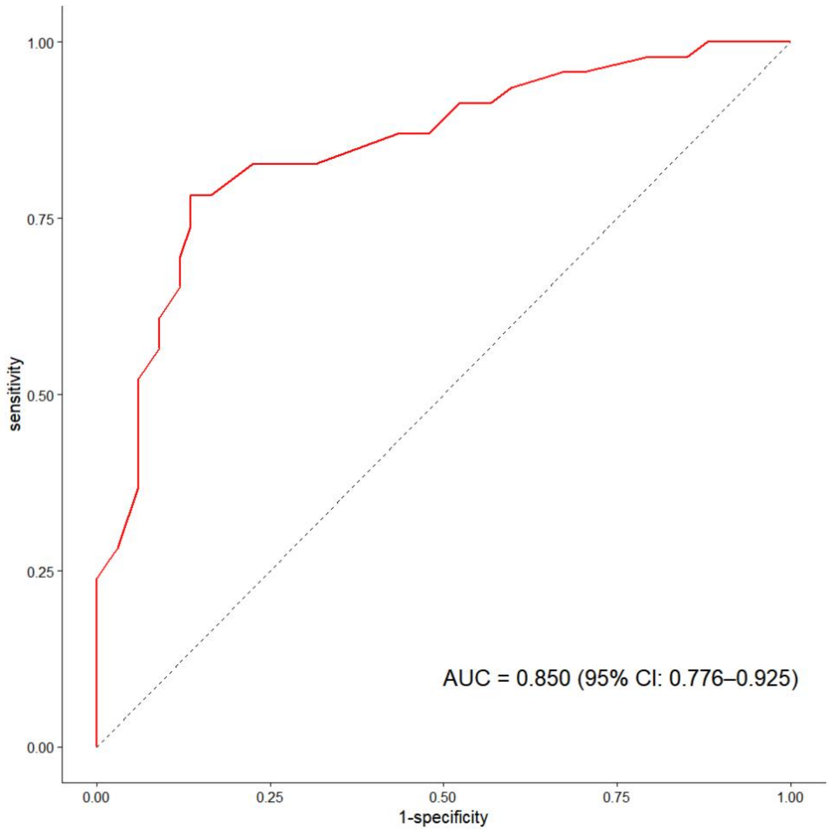

1.5 Supplementary Figure 3. The calibration curve of the model including PNI

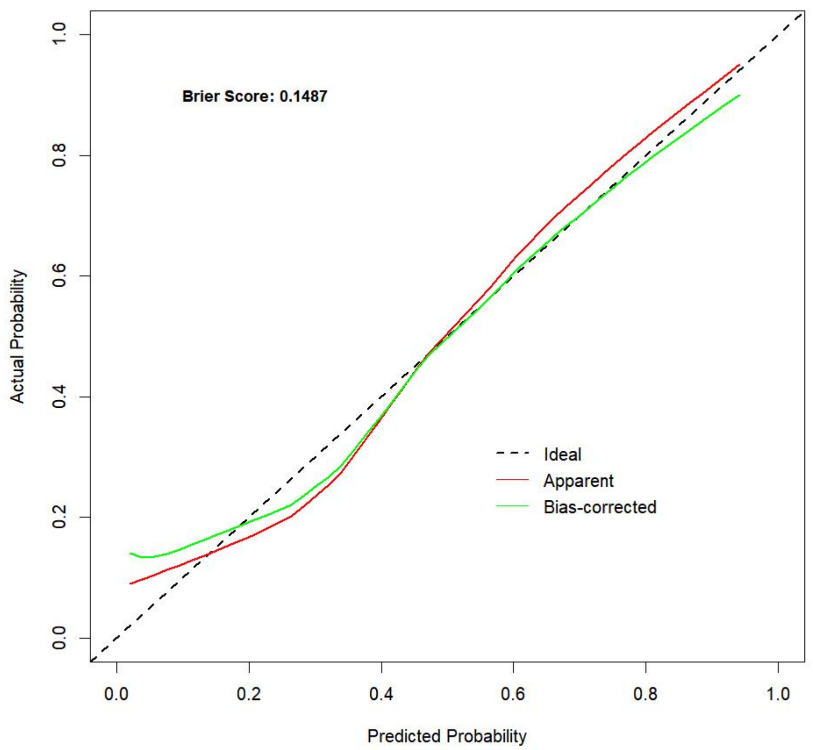
Brier Score: 0.1487
Ideal
Apparent
Bias-corrected
Actual Probability
Predicted Probability
0.0
0.2
0.4
0.6
0.8
1.0
